# Comparative safety assessment of nasogastric versus nasojejunal feeding initiated within 48 hours post-admission versus unrestricted timing in moderate or severe acute pancreatitis: a systematic review and meta-analysis

**DOI:** 10.1186/s12876-024-03290-z

**Published:** 2024-06-20

**Authors:** Ming Wang, Haiyan Shi, Qianqian Chen, Binbin Su, Xiaoyu Dong, Hui Shi, Shiping Xu

**Affiliations:** 1 Department of Gastroenterology, The Second Medical Center & National Clinical Research Center for Geriatric Diseases, Chinese PLA General Hospital, West Courtyard, No. 28 Fuxing Road, Haidian District, Beijing, 100853 China; 2 Department of Gastroenterology, the First Medical Center, General Hospital of the Chinese People’s Liberation Army, No. 28, Fu Xing Road, Hai Dian District, Beijing, 100853 China

**Keywords:** Acute pancreatitis, Nasogastric feeding, Nasojejunal feeding, Meta-analysis, Complications

## Abstract

**Background:**

The primary objective of this study is to comparatively assess the safety of nasogastric (NG) feeding versus nasojejunal (NJ) feeding in patients with acute pancreatitis (AP), with a special focus on the initiation of these feeding methods within the first 48 h of hospital admission.

**Methods:**

Studies were identified through a systematic search in PubMed, EMbase, Cochrane Central Register of Controlled Trials, and Web of Science. Four studies involving 217 patients were included. This systematic review assesses the safety and efficacy of nasogastric versus nasojejunal feeding initiated within 48 h post-admission in moderate/severe acute pancreatitis, with a specific focus on the timing of initiation and patient age as influential factors.

**Results:**

The results showed that the mortality rates were similar between NG and NJ feeding groups (RR 0.86, 95% CI 0.42 to 1.77, *P* = 0.68). Significant differences were observed in the incidence of diarrhea (RR 2.75, 95% CI 1.21 to 6.25, *P* = 0.02) and pain (RR 2.91, 95% CI 1.50 to 5.64, *P* = 0.002) in the NG group. The NG group also showed a higher probability of infection (6.67% vs. 3.33%, *P* = 0.027) and a higher frequency of multiple organ failures. Subgroup analysis for early intervention (within 48 h) showed a higher risk of diarrhea in the NG group (RR 2.80, *P* = 0.02). No significant differences were found in the need for surgical intervention, parenteral nutrition, or success rates of feeding procedures.

**Conclusion:**

This meta-analysis highlights the importance of considering the method and timing of nutritional support in acute pancreatitis. While NG feeding within 48 h of admission increases the risk of certain complications such as diarrhea and infection, it does not significantly impact mortality or the need for surgical intervention.

**Supplementary Information:**

The online version contains supplementary material available at 10.1186/s12876-024-03290-z.

## Background

Acute pancreatitis (AP), a prevalent digestive system disorder, is characterized by inflammation and functional impairment of the pancreas [1]. In adults, the most common etiologies of AP are gallstones and alcohol, accounting for a significant proportion of cases [2]. These factors contribute to the pathophysiological process leading to pancreatic inflammation and injury. Gallstones can obstruct the pancreatic duct, leading to pancreatic enzyme activation and inflammation, while alcohol contributes to AP through direct toxic effects on the pancreas and induction of oxidative stress [3]. The management of AP has evolved significantly over the years, with early enteral nutrition (EN) emerging as a pivotal aspect due to its potential to mitigate pancreatic stimulation, foster the recovery of intestinal barrier functions, and reduce the risk of complications [4].

The management of acute pancreatitis (AP) has been a subject of extensive research and clinical debate, with particular focus on the optimal timing and method of nutritional intervention. The concept of “early” in the context of AP management is not universally defined, but it generally refers to interventions initiated soon after the onset of symptoms or hospital admission. The definition of “early” is crucial as it influences the therapeutic approach and potentially the outcomes in AP patients. While there is no universally accepted time frame for what constitutes early intervention, a growing body of evidence suggests that nutritional intervention within the first 48 h of hospital admission can be particularly beneficial [5–8]. This 48-hour window is considered critical for several reasons. Firstly, it aligns with the early phase of AP, where interventions can potentially alter the disease course. Secondly, initiating enteral nutrition (EN) within this period may help in mitigating the systemic inflammatory response, which is often exacerbated in AP [9]. Early EN has been associated with reduced rates of infection, shorter hospital stays, and overall improved outcomes in AP patients [10]. Studies support the initiation of enteral nutrition within 48 h of acute pancreatitis onset, highlighting its benefits in reducing hospital mortality, length of stay, and pancreatic infection incidence [11–13].

The rationale behind early enteral nutrition (EN) in AP is grounded in its potential to reduce pancreatic stimulation, support the recovery of intestinal barrier function, and decrease the risk of complications associated with delayed feeding [14]. However, the choice between nasogastric (NG) and nasojejunal (NJ) feeding routes during this crucial early phase remains a contentious issue. NG feeding, traditionally the more common approach, has been challenged by the hypothesis that NJ feeding might be more effective in minimizing pancreatic stimulation due to its ability to bypass the pancreas more directly [15]. Despite the theoretical advantages of NJ feeding, empirical evidence remains inconclusive. Some studies have reported no significant differences between NG and NJ feeding in terms of nutritional support efficacy, complication rates, and length of hospital stay in severe AP cases [16]. However, concerns about gastric feeding intolerance (GFI), particularly in patients with more severe disease, have been raised [17].

Given the lack of consensus and clear guidelines on the optimal early feeding strategy in AP, our meta-analysis aims to provide a comprehensive comparison of NG versus NJ feeding within the first 48 h of hospital admission. By systematically analyzing available data, this study seeks to offer evidence-based recommendations for clinicians, facilitating informed decision-making in the early nutritional management of AP patients.

## Methods

### Data sources and search strategy

Following the PRISMA (Preferred Reporting Items for Systematic Reviews and Meta-Analyses) guidelines, we reviewed studies published in four databases: PubMed, EMbase, the Cochrane Central Register of Controlled Trials, and Web of Science. To identify relevant studies, we also examined the references of these articles. The search terms included “Pancreatitis,” “NG feeding,” “Nasojejunal feeding,” and “mortality,” used in various combinations. We excluded case reports, review articles, and non-randomized controlled trials (RCTs), limiting the language to English. The detailed search strategy was provided in Supplementary Table S1.

### Study selection

The following criteria were used to select studies suitable for meta-analysis: (1) RCTs. (2) Population: Patients with AP. (3) NG or NJ feeding initiated within the first 72 h of admission. (4) Studies must report at least one primary or secondary outcome. (5) Only English language articles were considered. The primary outcome was overall mortality, while secondary outcomes included organ failure, length of hospital stay, complications, infection rates, surgical intervention, requirement for parenteral nutrition, and the success rate of the procedure. Studies included in the meta-analysis were those in which enteral feeding (NG or NJ) was initiated within the first 72 h post-admission, specifically categorized into two subgroups: less than 48 h and 48 to 72 h.

### Data extraction and quality assessment

Data extraction and quality assessment were conducted by two independent reviewers using a structured data abstraction form, achieving high inter-observer consistency. Discrepancies were resolved through consensus or consultation with a third author. We extracted information including the authors’ names, article titles, journals of publication, countries and years of the studies, methodological variables, and clinical outcomes. The risk of bias in the included studies was assessed using the risk of bias assessment tool developed by the Cochrane Collaboration [18].

### Statistical analysis

Statistical analyses were conducted using RevMan 5.3 software. Outcomes were obtained either through direct extraction or indirect calculation. For binary data, risk ratios (RR) and their 95% confidence intervals (CI) were calculated, while for continuous variables, standardized mean differences (SMD) and their 95% CI were computed. Heterogeneity among studies was quantified using the I² statistic. Results were graphically represented using forest plots, and funnel plots were created to detect potential publication bias. Subgroup analyses were conducted to further explore statistically significant factors to reduce heterogeneity. These analyses primarily focused on comparing the impact of patient age and early intervention (within 48 h of admission) on the safety and efficacy of the two feeding methods. This research is registered with the International Prospective Register of Systematic Reviews (PROSPERO), number CRD42023485989.

## Results

**Fig. 1:**
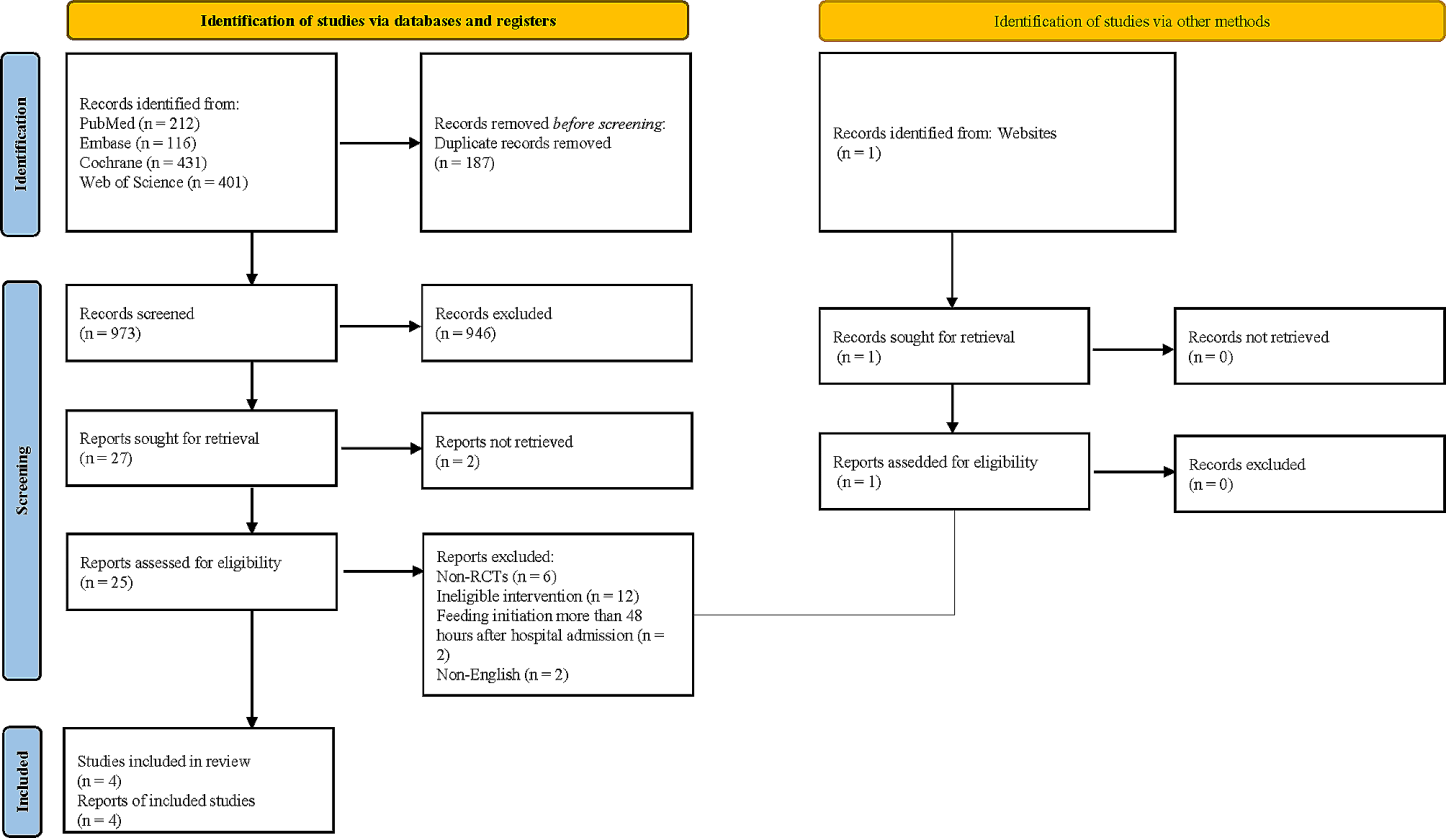
Flow chart of study selection

**Fig. 2:**
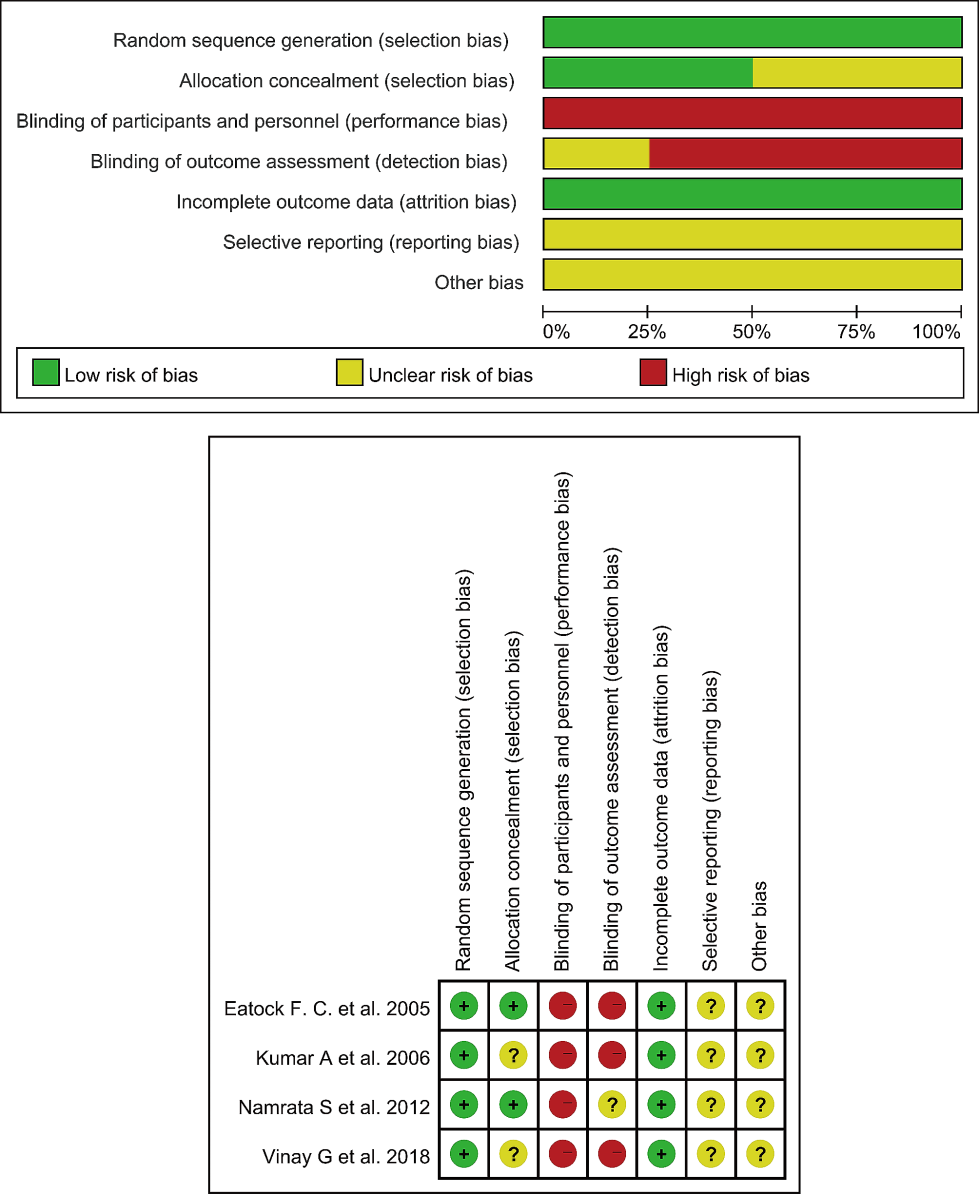
Risk of bias assessment chart of the included studies

Initially, our systematic search across multiple databases yielded a total of 1160 studies. After applying strict inclusion criteria based on the study design, population, and outcomes of interest, only four RCTs were deemed eligible for inclusion in our meta-analysis (Table 1). The RCTs we included involving a total of 217 patients, with 112 in the NG Feeding Group and 105 in the NJ Feeding Group [16, 19–21]. The specific study selection process was depicted in Fig. 1. Among all patients, there was a higher proportion of males, with an average age exceeding 35 years, and most were administered NG tube feeding within 48 h of hospital admission. The risk of bias in the included studies was presented in Fig. 2. The results indicated that none of the studies blinded participants and personnel, and three studies did not blind outcome assessment, leading to a high risk of performance and detection bias.

**Table 1:** General characteristics of the included studies

Study, Year	Country	Group	Number of patients	Age (years)	Gender (M/F)	Severity of Disease	Etiology, *n*(%)
Eatock et al. 2005​ [16]	Scotland	NG	27	Median age: 63 (IQR: 47–74)	14/13	moderate or severe	Gallstones: 16 (59.26) Alcohol:6 (22.22) Hereditary: 1 (3.70) Hyperparathyroidism: 1 (3.70) Idiopathic: 3 (11.11)
		NJ	22	Median age: 58 (IQR: 48–64)	12/10	moderate or severe	Gallstones: 16 (72.73) Alcohol:6 (27.27) Hereditary: 0 (0) Hyperparathyroidism: 0 (0) Idiopathic: 0 (0)
Vinay et al. 2018​ [21]	India	NG	30	Mean age: 36.34 (range: 22–64)	30/0	NR	Alcohol: 21 (70.0) Biliary: 6(20.0) Others: 3(10.0)
		NJ	30	Mean age: 38.73 (range: 24–68)	28/2	NR	Alcohol: 25(83.3) Biliary: 4(13.3) Others: 1(3.3)
Kumar et al. 2006 [19]​	India	NG	16	Mean ± SD: 43.25 ± 12.76	14/2	severe	Gallstones: 7 (43.8) Alcohol: 4 (25.0) Gallstones plus alcohol: 1 (6.3) Idiopathic: 4 (25.0)
		NJ	14	Mean ± SD: 35.57 ± 12.53	13/1	severe	Gallstones: 4 (28.6) Alcohol: 4 (28.6) Gallstones + alcohol: 1 (7.1) Idiopathic: 5 (35.7)
​Singh et al. 2012​ [20]	India	NG	39	Mean ± SD: 39.1 ± 16.7	28/11	severe	Gallstones 12 (30.8) Alcohol 12 (30.7) Idiopathic 9 (23.1) Others 6 (15.4)
		NJ	39	Mean ± SD: 39.7 ± 12.3	25/14	severe	Gallstones 21 (53.9) Alcohol 10 (25.6) Idiopathic 7 (17.9) Others 1 (2.6)

NR: Not reported

### Primary outcome

#### Mortality

**Fig. 3:**
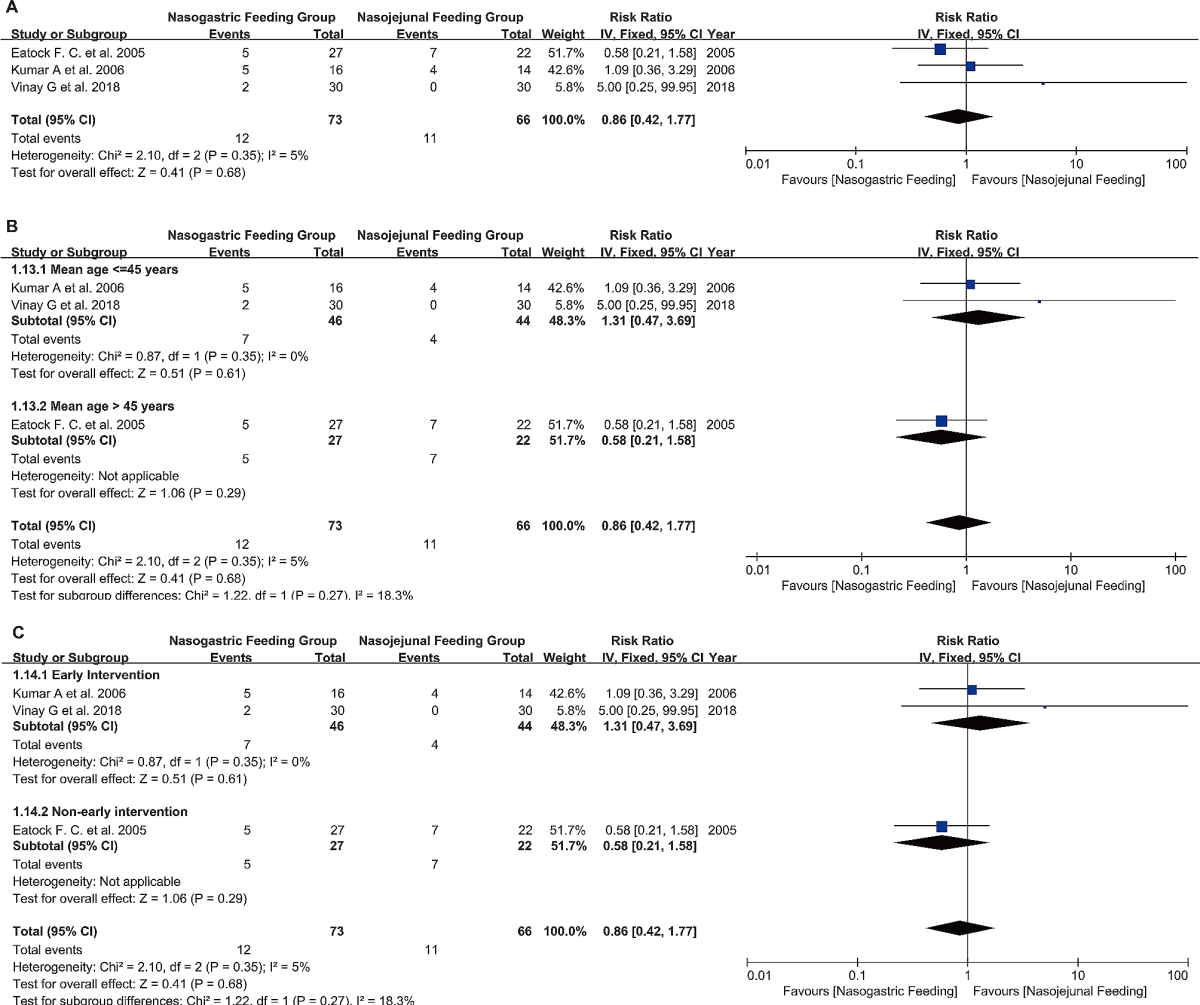
Forest plots of mortality and subgroup analyses **A**: Mortality comparison; **B**: Subgroup analysis of age-related mortality; **C**: Subgroup analysis of mortality related to time of nasogastric tube feeding

Mortality data were extracted from three of the included RCTs, 12 patients in the NG Feeding Group and 11 in the NJ feeding Group died. The forest plot results indicated similar mortality rates between the two groups, with low heterogeneity (RR 0.86, 95% CI 0.42 to 1.77, I²=5%, *P* = 0.68) (see Fig. 3A). The funnel plot revealed no evidence of publication bias in these studies (Figure S1 A).

##### Mean age ≤ 45 years vs. Mean age > 45 years

Subgroup analysis was conducted based on patient age. The RR was 1.31 in the group with an average age ≤ 45 years and 0.58 in the group with an average age > 45 years. These results indicate that age is not a significant factor affecting mortality (*P* = 0.27) (Fig. 3B).

##### Early intervention vs. varied-timing intervention

Subgroup analysis was performed based on the timing of NG tube feeding initiation in patients. The RR was 1.31 in the early intervention patients (within 48 h of admission) compared to interventions initiated at varied timings. These results suggest that the timing of intervention is not a significant factor affecting mortality (*P* = 0.27) (Fig. 3C).

### Secondary outcomes

#### Organ failure

In one study, organ failure was compared between the two groups of patients. The results indicated similar severity of organ failure in both groups, with over 70% of patients experiencing organ failure. In the NG Feeding Group, 76.67% (23 out of 30) of patients experienced multiple organ failures compared to 70% (21 out of 30) of patients in the NJ Feeding Group (*P* = 0.05) [21].

#### Length of stay

Among the four studies, hospital stay data from two could not be converted and thus were not included in the analysis. In the remaining two studies, the length of hospital stay was similar between the two groups, but with high heterogeneity (RR 0.56, 95% CI -0.97 to 2.10, I²=91%, *P* = 0.47) (Figure S2). The funnel plot indicated no publication bias in these studies (Figure S1 B). Two studies reported on the patients’ stay in the ICU. Eatock et al. [16] found that approximately one-quarter of patients in both groups were admitted to the respiratory intensive care unit during their hospital stay. The study by Vinay G et al. [21] found a higher number of patients in the NG Feeding Group admitted to the ICU compared to the NJ Feeding Group (*P* = 0.04).

#### Complication

**Fig. 4:**
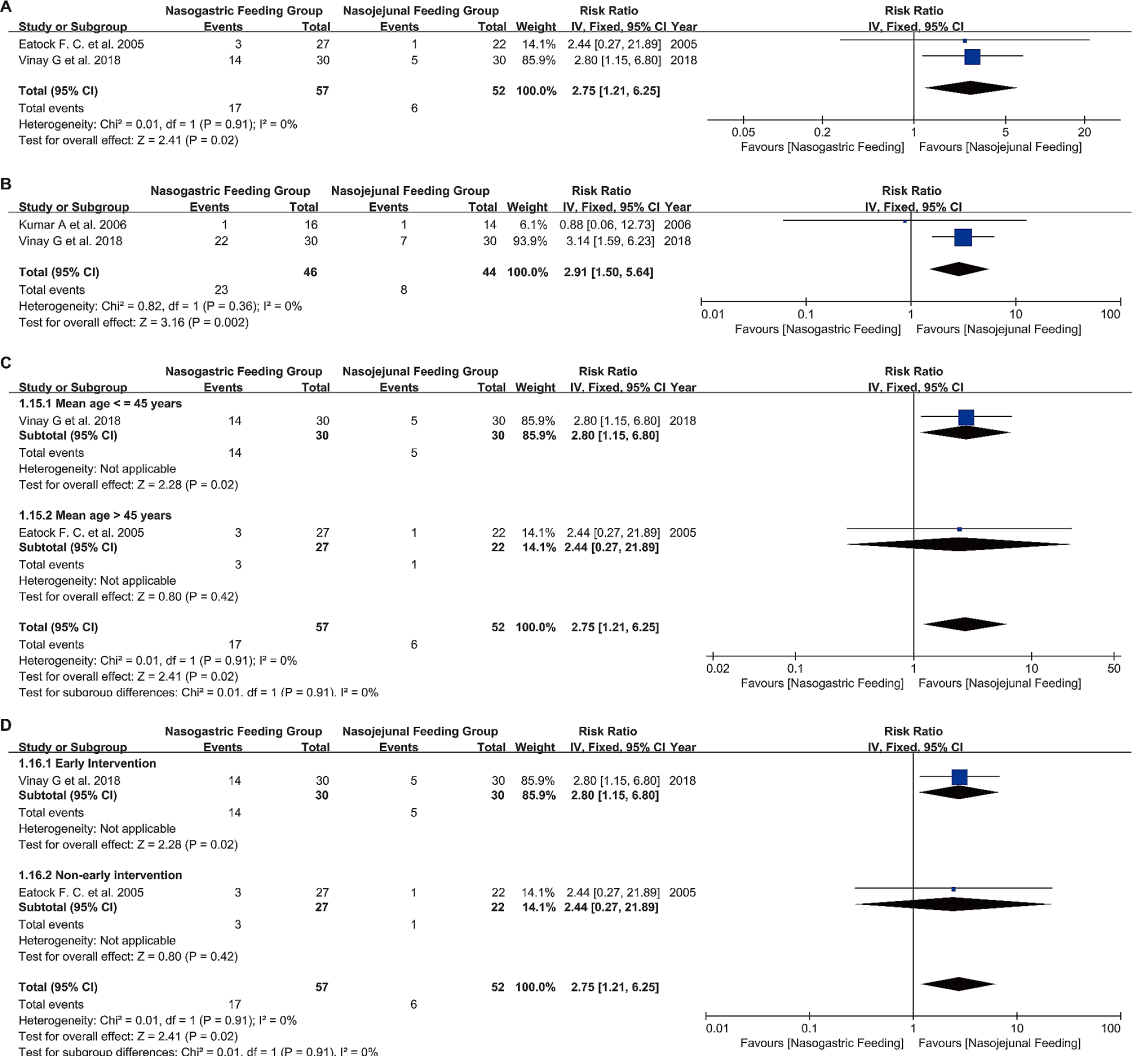
Forest plots of complications and subgroup analyses **A**: Incidence of diarrhea; **B**: Incidence of pain; **C**: Subgroup analysis of the incidence of age-related diarrhea; **D**: Subgroup analysis of the incidence of diarrhea associated with time of nasogastric tube feeding

Complications were reported in three of the included studies, primarily consisting of diarrhea and pain. The forest plot results indicated that patients in the NG Feeding Group had a higher frequency and risk of developing diarrhea (RR 2.75, 95% CI 1.21 to 6.25, I²=0%, *P* = 0.02) (Fig. 4A) and pain (RR 2.91, 95% CI 1.50 to 5.64, I²=0%, *P* = 0.002) (Fig. 4B) compared to the NJ Feeding Group. The funnel plot did not reveal any publication bias (Figures S1 C-D). The study by Vinay G et al. [21] reported on complications related to pancreatitis, finding that patients in the NG Feeding Group were likely to develop Acute Fluid Collection.

##### Mean age ≤ 45 years vs. Mean age > 45 years

Subgroup analysis was conducted based on patient age. In the subgroup with an average age ≤ 45 years, the RR for diarrhea in the NG Feeding Group was 4.38, indicating a higher risk of developing diarrhea in this group (*P* = 0.02). For the subgroup with an average age > 45 years, the RR was 2.63. These results suggest that age was not a significant factor in the occurrence of complications, but special attention should be paid to the occurrence of diarrhea in patients aged ≤ 45 years (*P* = 0.70) (Fig. 4C).

##### Early intervention vs. varied-timing intervention

Subgroup analysis was performed based on the timing of NG tube feeding initiation in patients. In the early intervention group (feeding initiated within 48 h of admission), the NG Feeding Group had a RR of 2.80 for developing diarrhea, indicating a higher risk in this group (*P* = 0.02). In the varied-timing intervention group, the RR was 2.44. These results suggest that the timing of NG tube feeding initiation is not a significant factor in the occurrence of complications, but the first 48 h after starting feeding are particularly important (*P* = 0.91) (Fig. 4D).

### Rate of infection

**Fig. 5:**
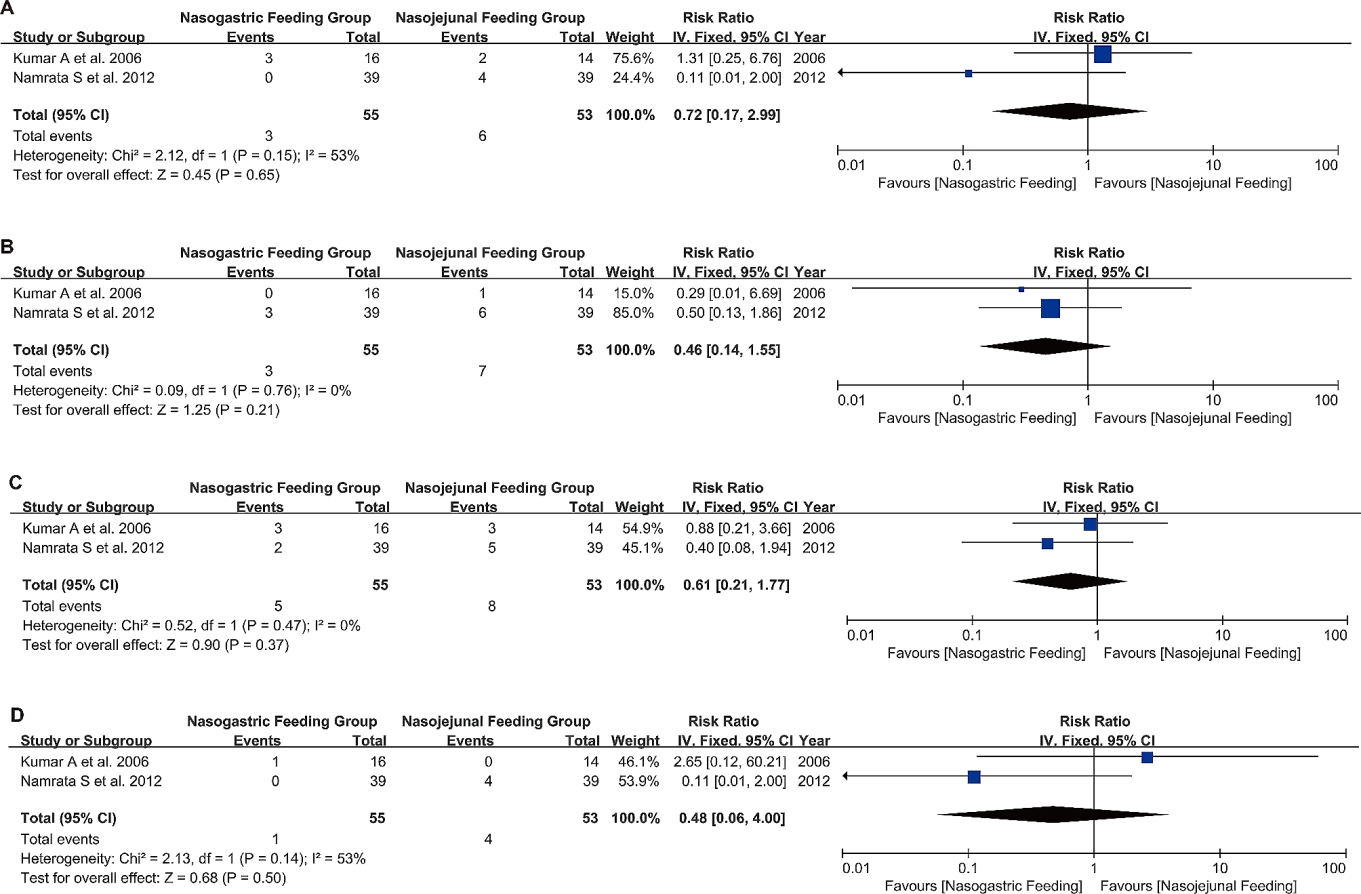
Forest plot of infection detection methods **A**: Blood cultures were positive; **B**: Tracheal aspirate; **C**: Pancreatic aspirate; **D**: Biliary tract culture

In the study by Vinay G et al. [21], the incidence of infections in patients was relatively low for both NG and NJ Feeding, but the NG Feeding Group had a higher probability of infection (6.67% vs. 3.33%, *P* = 0.027). Analysis of various methods used for detecting infections, including blood culture positive (RR 0.72, 95% CI 0.17 to 2.99, I²=53%, *P* = 0.65) (Fig. 5A), tracheal aspirate (RR 0.46, 95% CI 0.14 to 1.55, I²=0%, *P* = 0.21) (Fig. 5B), pancreatic aspirate (RR 0.61, 95% CI 0.21 to 1.77, I²=91%, *P* = 0.37) (Fig. 5C), and bile culture (RR 0.48, 95% CI 0.06 to 4.00, I²=53%, *P* = 0.50) (Fig. 5D), showed similar efficacy and low heterogeneity. The funnel plot results also did not indicate any publication bias (Figures S2E-H).

### Surgical intervention

**Fig. 6:**
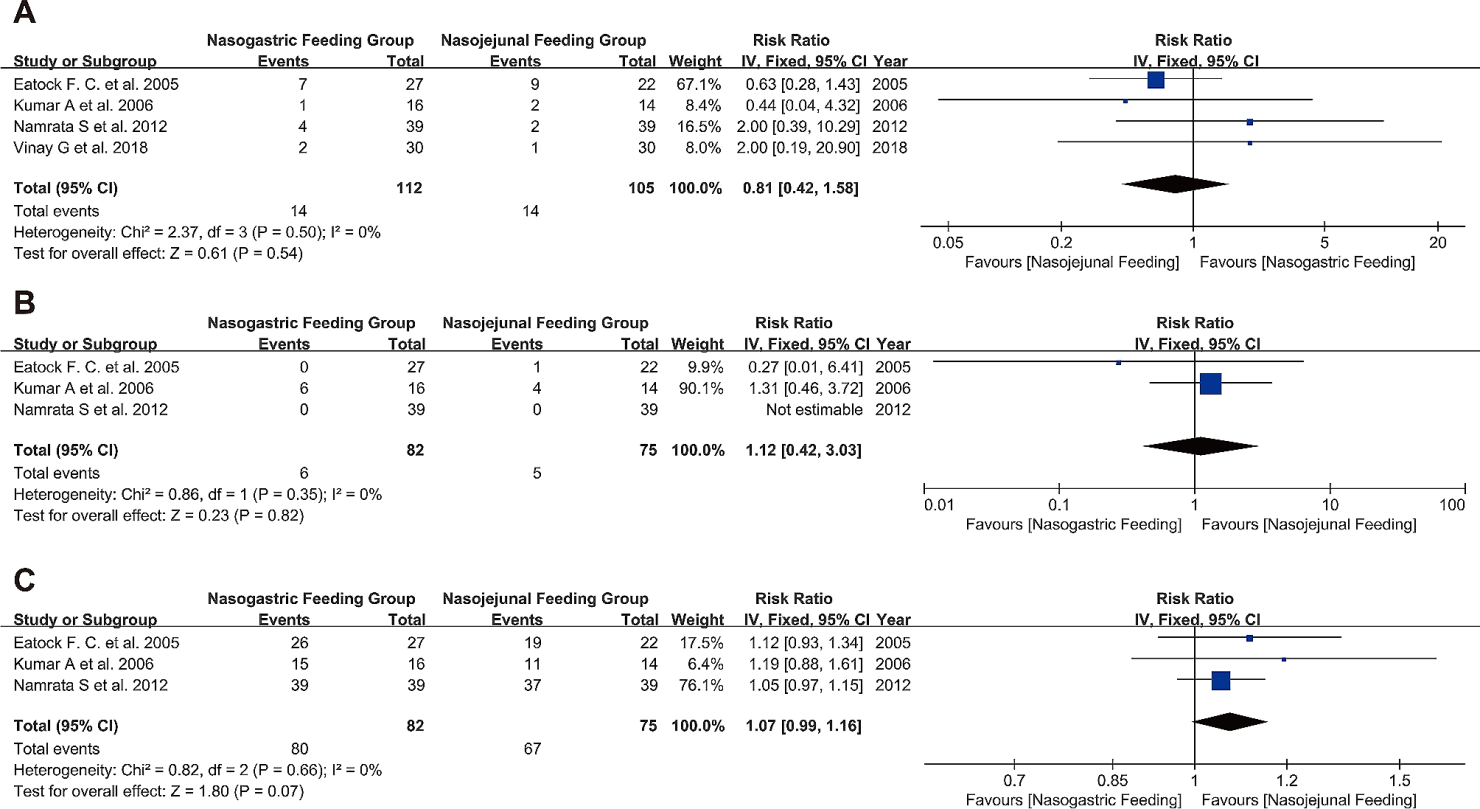
Forest plot of surgical intervention and parenteral nutrition requirements **A**: Comparison of demands for surgical intervention; **B**: Comparison of demands for parenteral nutrition interventions; **C**: Comparison of success rates of nasogastric tube feeding

In four trials, the need for surgical intervention among participants was reported. Out of a total of 217 patients, 28 underwent surgical intervention. The proportion of patients requiring surgery was similar in both groups, with low heterogeneity (RR 0.81, 95% CI 0.42 to 1.58, I²=0%, *P* = 0.54) (Fig. 6A). The funnel plot results also did not indicate any publication bias (Figure S3 A).

#### Early intervention vs. varied-timing intervention

In the subgroup analysis based on the timing of NG tube feeding initiation, the early intervention group had a RR of 1.36, while the varied-timing intervention group had a RR of 0.63. These results suggested that the timing of intervention was not a significant factor affecting the need for surgical intervention (*P* = 0.29) (Figure S4A).

### Requirement for parenteral nutrition

In three studies, out of 157 patients, 11 underwent parenteral nutrition intervention. The forest plot results showed no significant difference between the two groups in terms of receiving parenteral nutrition (RR 1.12, 95% CI 0.42 to 3.03, I²=0%, *P* = 0.82) (Fig. 6B). The funnel plot results also did not indicate any publication bias (Figure S3 B).

#### Early intervention vs. varied-timing intervention

In the subgroup analysis based on the timing of NG tube feeding initiation, the early intervention group had a RR of 1.31, while the varied-timing intervention group had a RR of 0.27. These results suggested that the timing of intervention was not a significant factor in the need for parenteral nutrition (*P* = 0.35) (Figure S4B).

### Success rate of the procedure

In three studies, 80 of 82 patients who received NG tube feeding were successful; 67 of 75 patients who received NJ tube feeding were successful. The forest plot results showed that the success rates of feeding were similar between the two groups, with low heterogeneity (RR 1.07, 95% CI 0.99 to 1.16, I²=0%, *P* = 0.07) (Fig. 6C). The funnel plot results also did not indicate any publication bias (Figure S3 C).

#### Early intervention vs. varied-timing intervention

In the subgroup analysis based on the timing of NG feeding initiation, the early intervention group had a RR of 1.06, while the varied-timing intervention group had a RR of 1.12. These results suggested that the timing of intervention was not a significant factor affecting the success rate of the procedure (*P* = 0.64) (Figure S4C).

## Discussion

Our meta-analysis provides a comprehensive evaluation of the comparative safety and efficacy of NG versus NJ feeding in AP patients, particularly focusing on early intervention within 48 h of hospital admission. Our findings, derived from four RCTs [16, 19–21] encompassing 217 patients, offer critical insights into the management of acute pancreatitis, shedding light on the optimal feeding strategies in AP, with a particular emphasis on the critical 48-hour window post-admission. It is important to note that three out of the four studies included in this meta-analysis primarily involve patients with severe AP, while the study by Vinay et al. [21]. includes patients across a broader spectrum of disease severity. This distinction is crucial as it predominantly reflects the outcomes of severe AP management and could influence the generalizability of our results.

The primary outcome of our study, overall mortality, showed no significant difference between the NG and NJ feeding groups​​​​. This finding contradicts the common presumption that NJ feeding, due to its reduced risk of aspiration and theoretically more physiological delivery of nutrients, might confer a mortality benefit. In fact, studies have shown that NG feeding is as good as NJ feeding in patients with objectively graded severe acute pancreatitis, with no significant differences in mortality [22]. Studies have shown that age is a significant factor in mortality in acute pancreatitis, with higher mortality rates observed in older patients [23, 24]. However, our subgroup analysis based on age further demonstrates that age is not a significant factor affecting mortality rates in AP patients undergoing either NG or NJ feeding. This observation is crucial for clinical practice, as it suggests that the choice of feeding route need not be influenced by patient age, which is a key consideration in managing AP.

Our analysis also delves into the timing of feeding initiation. The subgroup analyses comparing patients who all received early enteral nutrition (within 48 h of admission) with those where only a portion received early intervention revealed no significant difference in mortality rates or the success of the procedure. This result suggests that while early enteral nutrition is critical in AP management, the specific timing within the early phase may not be as pivotal as previously thought. Studies have shown that early enteral nutrition, particularly within the first 48 h, is associated with reduced mortality and infectious complications in severe acute pancreatitis patients [9, 25]. However, some studies have raised questions about the beneficial impact of the specific timing of early intervention on mortality [26, 27]. These findings are important for clinical practice, indicating that while early enteral nutrition is advantageous, flexibility in the timing of initiation within the early intervention window can still yield positive outcomes. This flexibility is particularly relevant in clinical settings where various factors can influence the timing of nutrition initiation.

In terms of secondary outcomes, organ failure rates were comparable between the two groups, though a higher incidence of multiple organ failures was noted in the NG feeding group in one study [21]​​. However, the length of hospital stay and ICU admission did not significantly differ between the groups, implying that the route of feeding does not substantially impact the outcomes. This finding is consistent with previous studies, which have shown that NG nutrition is as safe and effective as NJ nutrition in patients with severe acute pancreatitis, with no significant differences in hospital stay [22, 28, 29]. In terms of infection rates, our study found no significant differences between NG and NJ feeding. This challenges the prevailing assumption that NJ feeding, which theoretically reduces microbial translocation, might offer lower infection rates in patients with severe acute pancreatitis [30, 31]. These findings suggest that the route of enteral nutrition, whether NG or NJ, may not significantly influence the risk of infection in AP patients. The necessity for surgical intervention and parenteral nutrition was comparable across both feeding groups, indicating similar effectiveness in clinical management regardless of the feeding route.

Complications such as diarrhea and pain were more prevalent in the NG group. This finding aligns with the physiological understanding that NJ feeding, bypassing the stomach, might be less irritating and hence less likely to induce such complications. However, it’s important to note that studies have shown mixed results in this regard. Some research indicates that NJ feeding can significantly decrease the recurrence of bellyache and shorten the duration of treatment compared to NG route in acute pancreatitis patients [30]. A randomized trial by Casaer et al. [32] reported that the risk of diarrhea was higher with postpyloric feeding compared to parenteral nutrition (risk ratio 1.71, 95% CI 1.04–2.79). However, a meta-analysis by Elke et al. did not find a statistically significant difference in diarrhea between gastric and jejunal feeding tubes (RR 1.28, 95% CI 0.70–2.33) [33]. In regards to abdominal pain, a cohort study by Poulard et al. found no significant difference between NG and parenteral nutrition groups (NG 15% vs. TPN 13%, *P* = 0.83) [34]. However, the age-based subgroup analysis suggested that age is not a significant factor in the occurrence of these complications, though younger patients may require closer monitoring. More research may be needed to clarify feeding-related gastrointestinal side effects between enteral and parenteral routes. These findings suggest that while NJ feeding may theoretically reduce gastrointestinal irritation, the actual clinical impact on complication rates such as diarrhea and pain may not be as pronounced as expected. Furthermore, age analysis revealed no significant factor affecting mortality rates in AP patients undergoing either NG or NJ feeding, suggesting that the choice of feeding route need not be influenced by patient age.

Our study is not without limitations. The included RCTs demonstrated some degree of performance and detection bias, which could impact the reliability of the outcomes. Notably, the lack of blinding in the included studies introduces potential biases that could influence the outcomes. Due to the small number of included studies, we were limited from conducting a more detailed analysis of age-related effects. Additionally, the heterogeneity observed in secondary outcomes like hospital stay duration necessitates more uniform study designs in future research.

## Conclusion

Our meta-analysis concludes that that both NG and NJ feeding are viable options in the early management of acute pancreatitis, with no significant difference in mortality and other major clinical outcomes. The choice of feeding route should be individualized, taking into consideration patient-specific factors and clinical contexts. Future research should aim to enhance study design uniformity and focus on exploring patient-centered outcomes to address existing knowledge gaps. This evidence-based approach will be pivotal in optimizing AP management strategies in clinical practice.

### Electronic supplementary material

Below is the link to the electronic supplementary material.

Supplementary Material 1

Supplementary Material 2

Supplementary Material 3

Supplementary Material 4

Supplementary Material 5

## Data Availability

No datasets were generated or analysed during the current study.
