# Supplementary figures and images for: Comparative safety assessment of nasogastric versus nasojejunal feeding initiated within 48 hours post-admission versus unrestricted timing in moderate or severe acute pancreatitis: a systematic review and meta-analysis
Source: BMC Gastroenterol. 2024 Jun 20;24:207. doi: 10.1186/s12876-024-03290-z (PMC11188158; doi:10.1186/s12876-024-03290-z)

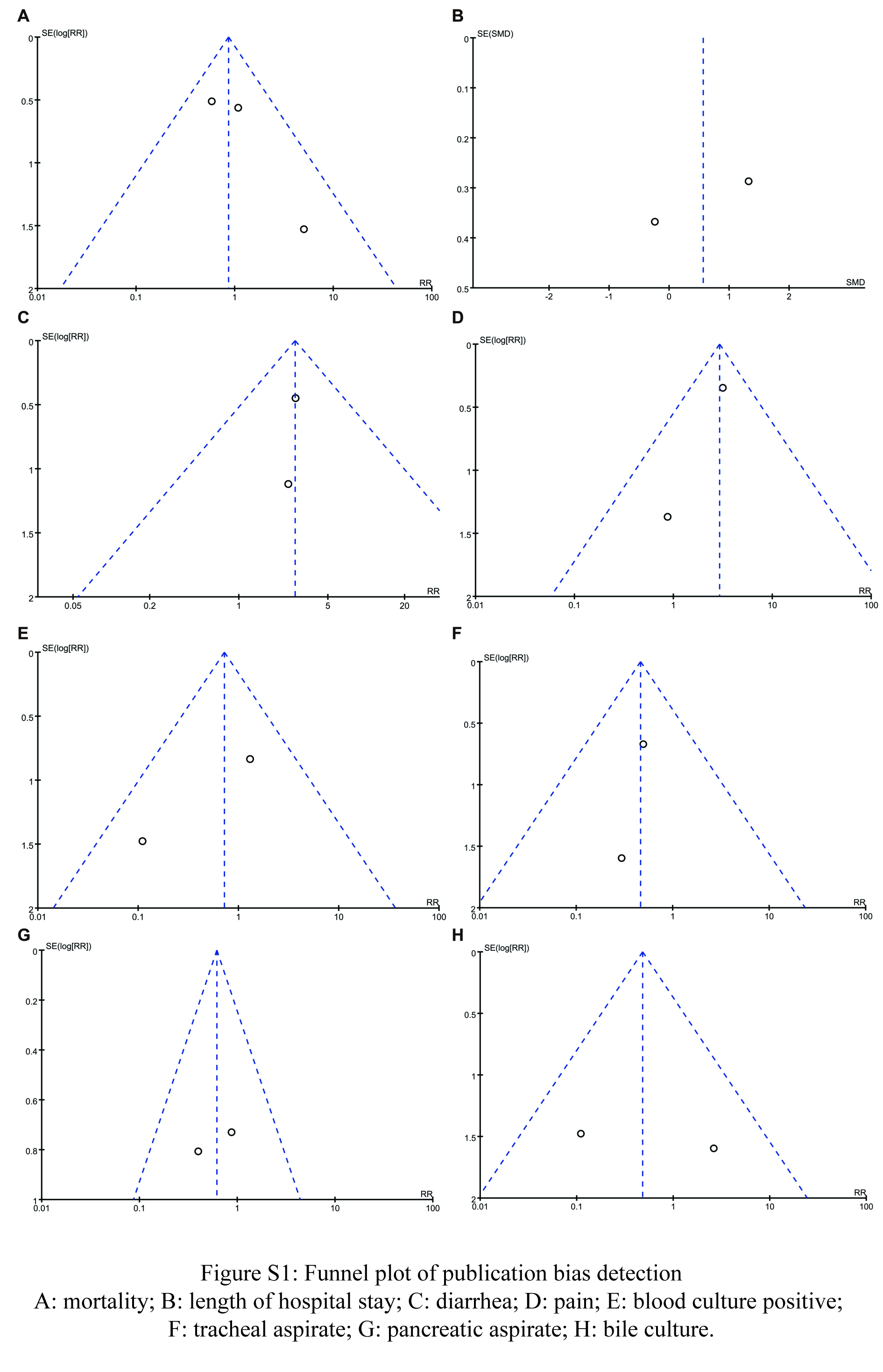

Supplement: Supplementary file 1 — Supplementary Material 1 [file 12876_2024_3290_MOESM1_ESM.tif]

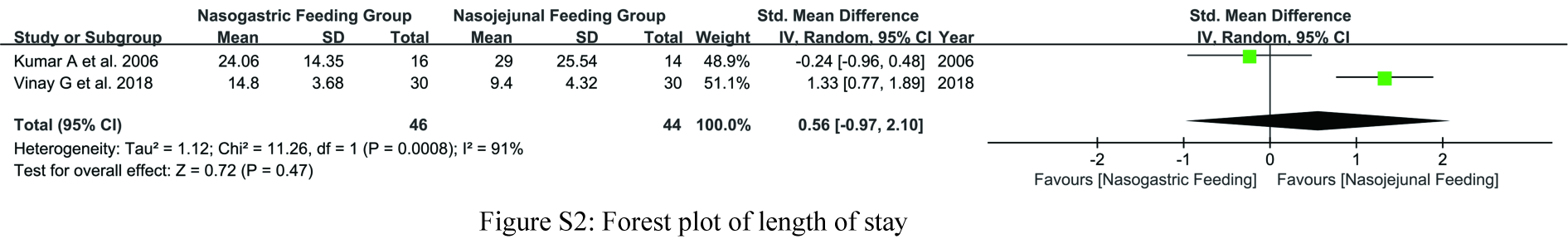

Supplement: Supplementary file 2 — Supplementary Material 2 [file 12876_2024_3290_MOESM2_ESM.tif]

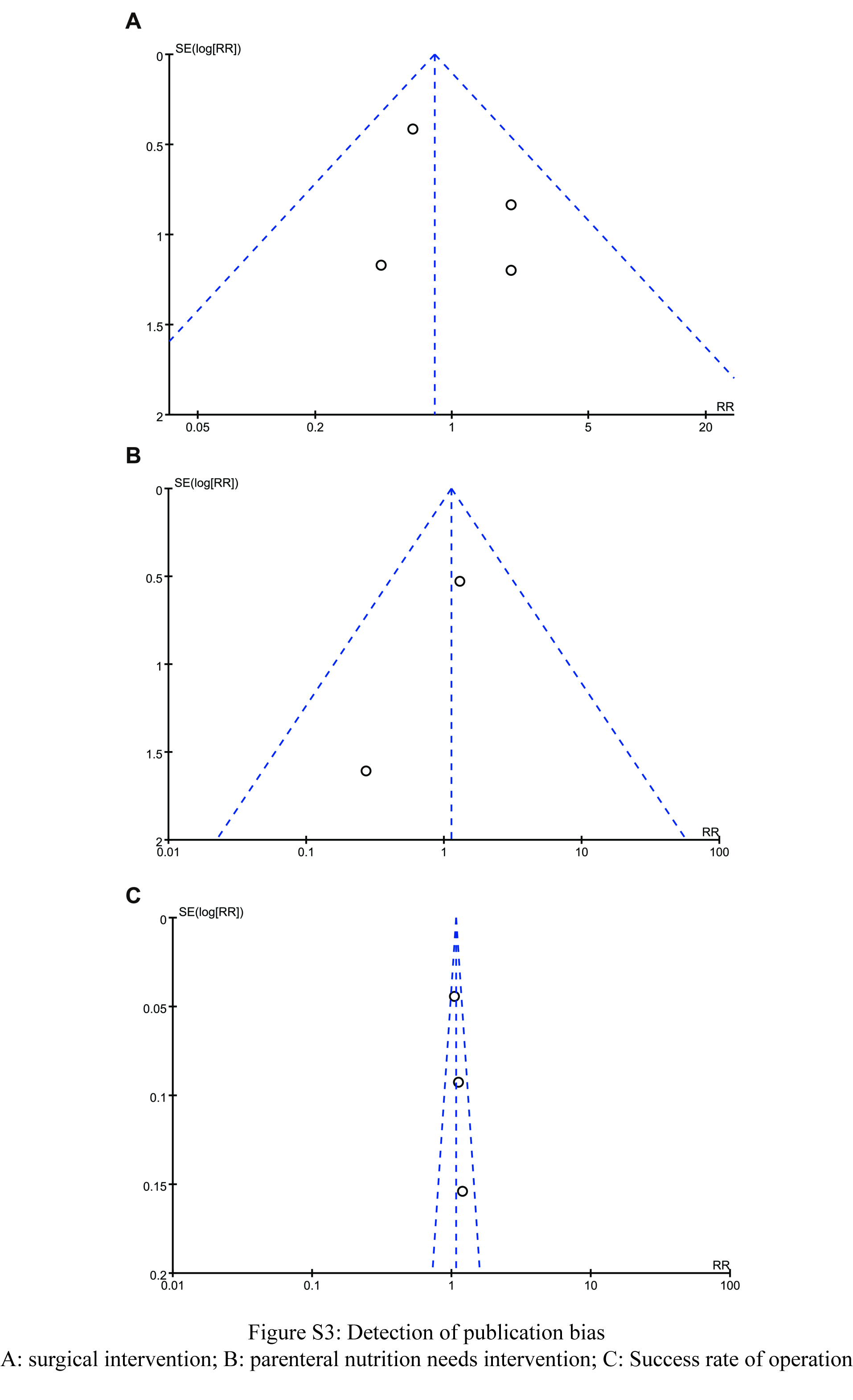

Supplement: Supplementary file 3 — Supplementary Material 3 [file 12876_2024_3290_MOESM3_ESM.tif]

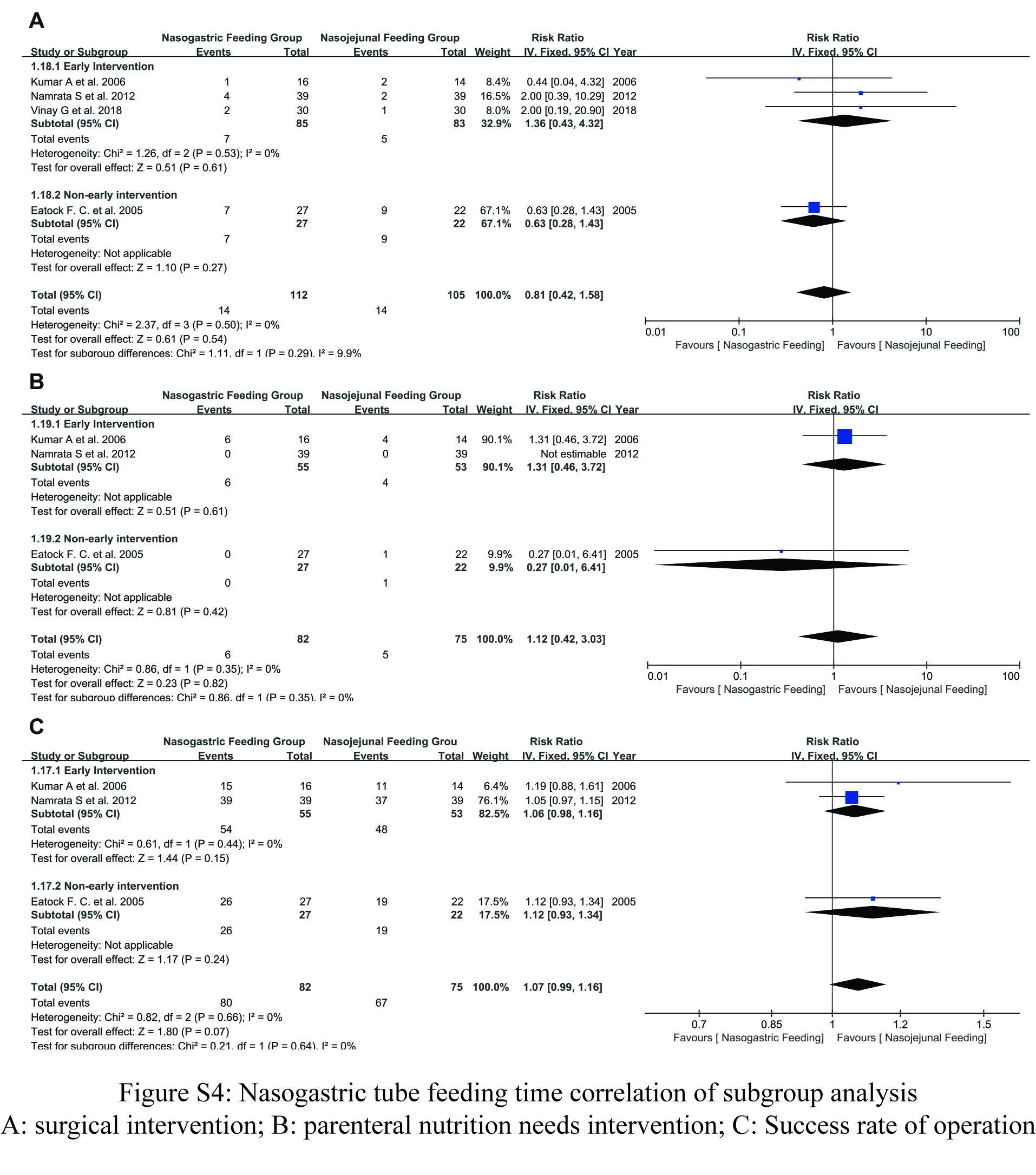

Supplement: Supplementary file 4 — Supplementary Material 4 [file 12876_2024_3290_MOESM4_ESM.tif]
