# Supplementary material for: Comparative safety assessment of nasogastric versus nasojejunal feeding initiated within 48 hours post-admission versus unrestricted timing in moderate or severe acute pancreatitis: a systematic review and meta-analysis
Source: BMC Gastroenterol. 2024 Jun 20;24:207. doi: 10.1186/s12876-024-03290-z (PMC11188158; doi:10.1186/s12876-024-03290-z)
Supplement: Supplementary file 5 — Supplementary Material 5 [file 12876_2024_3290_MOESM5_ESM.docx]

**Table S1. List of search terms in each database.**

| **Database** | **Set** | **Search terms** | **Results** |
| --- | --- | --- | --- |
| **PubMed** | #1 | "Pancreatitis"[MeSH] AND ("Acute"[Title/Abstract] OR "AP"[Title/Abstract]) AND ("early"[Title/Abstract] OR "initial"[Title/Abstract] OR "first 72 hours"[Title/Abstract] OR "onset"[Title/Abstract]) | 5,711 |
|  | #2 | "Enteral Nutrition"[MeSH] OR "Enteral Feeding"[Title/Abstract] OR "NG feeding"[Title/Abstract] OR "NE feeding"[Title/Abstract] OR "Nasogastric feeding"[Title/Abstract] OR "Nasogastric tube"[Title/Abstract] OR "Nasoenteric feeding"[Title/Abstract] OR "Nasoenteric tube"[Title/Abstract] OR "Nasoduodenal feeding"[Title/Abstract] OR "Nasoduodenal tube"[Title/Abstract] OR "Nasojejunal feeding"[Title/Abstract] OR "Nasojejunal tube"[Title/Abstract] OR "enteral tube feeding"[Title/Abstract] | 29,929 |
|  | #3 | "safety"[Title/Abstract] OR "complications"[Title/Abstract] OR "mortality"[Title/Abstract] OR "adverse events"[Title/Abstract] OR "adverse effects"[Title/Abstract] OR "risk"[Title/Abstract] OR "harm"[Title/Abstract] OR "morbidity"[Title/Abstract] OR "side effects"[Title/Abstract] OR "intolerance"[Title/Abstract] OR "feeding failure"[Title/Abstract] OR "gastrointestinal symptoms"[Title/Abstract] OR "risk factors"[Title/Abstract] | 5,114,781 |
|  | #4 | #1 AND #2 AND #3 | 212 |
| **Cochrane** | #1 | MeSH descriptor: [Pancreatitis, Acute] explode all trees | 1,907 |
|  | #2 | (Pancreatitis OR "Acute Pancreatitis" OR "early phase" OR "early onset" OR initial OR "within 72 hours" OR onset):ti,ab,kw (Word variations have been searched) | 150,725 |
|  | #3 | #1 OR #2 | 150,725 |
|  | #4 | MeSH descriptor: [Enteral Nutrition] explode all trees | 2,195 |
|  | #5 | ("Enteral Feeding" OR "NG feeding" OR "NE feeding" OR "Nasogastric feeding" OR "Nasogastric tube" OR "Nasoenteric feeding" OR "Nasoenteric tube" OR "Nasoduodenal feeding" OR "Nasoduodenal tube" OR "Nasojejunal feeding" OR "Nasojejunal tube" OR "enteral tube feeding"):ti,ab,kw | 3,965 |
|  | #6 | #4 OR #5 | 5,200 |
|  | #7 | MeSH descriptor: [Patient Safety] explode all trees | 1,530 |
|  | #8 | ("safety" OR "complications" OR "mortality" OR "adverse events" OR "risk" OR "harm" OR "morbidity" OR "side effects" OR "intolerance" OR "feeding failure" OR "gastrointestinal symptoms" OR "risk factors"):ti,ab,kw (Word variations have been searched) | 787,423 |
|  | #9 | #7 OR #8 | 787,423 |
|  | #10 | #3 AND #6 AND #9 in Trials | 431 |
| **Web of Science** | #1 | TS=(("Acute Pancreatitis" OR "AP") AND (early OR initial OR "first 72 hours" OR onset)) | 16,424 |
|  | #2 | TS=("Enteral Nutrition" OR "Enteral Feeding" OR "NG feeding" OR "NE feeding" OR "Nasogastric feeding" OR "Nasogastric tube" OR "Nasoenteric feeding" OR "Nasoenteric tube" OR "Nasoduodenal feeding" OR "Nasoduodenal tube" OR "Nasojejunal feeding" OR "Nasojejunal tube" OR "enteral tube feeding") | 23,410 |
|  | #3 | TS=(safety OR complications OR mortality OR "adverse events" OR "adverse effects" OR risk OR harm OR morbidity OR "side effects" OR intolerance OR "feeding failure" OR "gastrointestinal symptoms" OR "risk factors") | 7,023,770 |
|  | #4 | #1 AND #2 AND #3 | 401 |
| **Embase** | #1 | 'acute pancreatitis'/exp AND ("early phase":ti,ab OR "early onset":ti,ab OR "initial":ti,ab OR "within 72 hours":ti,ab) | 2,910 |
|  | #2 | 'enteral nutrition'/exp OR 'nasogastric feeding'/exp OR 'nasoenteric feeding'/exp OR "Enteral Feeding":ti,ab OR "NG feeding":ti,ab OR "NE feeding":ti,ab OR "Nasogastric feeding":ti,ab OR "Nasogastric tube":ti,ab OR "Nasoenteric feeding":ti,ab OR "Nasoenteric tube":ti,ab OR "Nasoduodenal feeding":ti,ab OR "Nasoduodenal tube":ti,ab OR "Nasojejunal feeding":ti,ab OR "Nasojejunal tube":ti,ab OR "enteral tube feeding":ti,ab | 73,799 |
|  | #3 | 'patient safety'/exp OR 'complication'/exp OR "safety":ti,ab OR "complications":ti,ab OR "mortality":ti,ab OR "adverse events":ti,ab OR "adverse effects":ti,ab OR "risk":ti,ab OR "harm":ti,ab OR "morbidity":ti,ab OR "side effects":ti,ab OR "intolerance":ti,ab OR "feeding failure":ti,ab OR "gastrointestinal symptoms":ti,ab OR "risk factors":ti,ab | 8,478,987 |
|  | #4 | #1 AND #2 AND #3 | 116 |
